# Reassessment of somatostatin receptor SST4 expression in bronchopulmonary and gastroenteropancreatic neuroendocrine neoplasms using the novel rabbit monoclonal anti-human SST4 antibody 7H49L61

**DOI:** 10.1038/s41598-022-19014-w

**Published:** 2022-08-30

**Authors:** Blanca Ehms, Daniel Kaemmerer, Jörg Sänger, Stefan Schulz, Amelie Lupp

**Affiliations:** 1 Institute of Pharmacology and Toxicology, Jena University Hospital, Friedrich Schiller University Jena, Drackendorfer Str. 1, 07747 Jena, Germany; 2 Department of General and Visceral Surgery, Zentralklinik Bad Berka, Bad Berka, Germany; 3 Laboratory of Pathology and Cytology Bad Berka, Bad Berka, Germany

**Keywords:** Cancer, Biomarkers, Endocrinology, Molecular medicine, Oncology

## Abstract

Somatostatin receptors SST1, SST2, and SST5 are overexpressed in neuroendocrine neoplasms (NENs), but little is known about SST4 expression in NENs because of a lack of specific monoclonal antibodies. We recently developed and thoroughly characterised a rabbit monoclonal anti-human SST4 antibody, 7H49L61, and showed that it is well suited for identifying SST4 expression in routine pathology samples. The present study aimed to re-evaluate SST4 expression in a large set of NEN samples using this antibody. For this purpose, we assessed SST4 expression in 722 formalin-fixed, paraffin-embedded NEN samples from 274 patients by immunohistochemistry using the novel antibody 7H49L61. The immunostaining was semiquantitatively evaluated using the 12-point immunoreactivity score (IRS), and the results were correlated with clinicopathological data. SST4 was detected in 39.3% of all NENs, but with a median IRS of 2.0, its expression intensity was negligible overall. In all cases, both cytoplasmic and membraneous staining was observed. SST4 expression was somewhat higher in bronchopulmonary NEN (BP-NEN) than in gastroenteropancreatic NEN (GEP-NEN) but still very low. SST4 expression positively correlated with favourable patient outcomes in BP-NEN but had a positive association with Ki-67 index or tumour grading and a negative interrelationship with overall survival in GEP-NEN. In conclusion, unlike that of other SST subtypes, SST4 expression in both BP-NEN and GEP-NEN is negligible and of no diagnostic or therapeutic relevance.

## Introduction

With an incidence of 2.5–7 per 100,000 people per year, neuroendocrine neoplasms (NENs) are rare malignancies. However, in recent decades, the incidence of these tumours has increased, which might be due in part to improved diagnostic modalities^1–5^. NENs arise from the neuroendocrine cells of the diffuse endocrine system and can develop in almost every organ and tissue, but they are most often found in the gastrointestinal tract, the pancreas, or the lung^6^. Based on histopathological criteria, mitotic count, and Ki-67 index, according to the current World Health Organization classification NENs are generally classified into well-differentiated G1, G2, or G3 tumours and poorly differentiated G3 carcinomas with small-cell or large-cell morphology^4,6–8^. Bronchopulmonary NENs (BP-NENs) are also categorised into four major entities: grade 1 typical carcinoids (TCs), grade 2 atypical carcinoids (ACs), and grade 3 small-cell lung carcinomas (SCLCs) or large-cell neuroendocrine carcinomas of the lung (LCNECs)^6,8,9^. G1 and G2 gastroenteropancreatic NENs (GEP-NENs) overexpress somatostatin receptors (SSTs), especially SST2 and SST5 (e.g.,^10–19^), which enables functional imaging of these tumours by scintigraphy or positron emission tomography/computed tomography as well as pharmacotherapy or peptide-receptor radionuclide therapy with (radiolabelled) somatostatin analogues, predominantly targeting SST2 or SST5^20^. SST expression is generally lower in BP-NEN than in GEP-NEN and has distinct patterns between the two tumour types. In GEP-NEN, SST2 is most highly expressed, followed by SST5 (e.g.,^10–19^), whereas in BP-NEN, SST1 expression seems to prevail (e.g.,^21–24^). In contrast to GEP-NEN, SST-based diagnostics or therapy have not been established as routine modalities for BP-NEN so far^25–27^.

Compared with the other SSTs, for which highly specific and well characterised monoclonal antibodies have been available for many years, little is known about SST4 expression in NEN, especially BP-NEN. Existing knowledge is based on mRNA expression analyses, receptor autoradiography studies, and immunostaining, mostly with poorly characterised polyclonal antibodies. Overall, results are inconsistent, and reported SST4 positivity rates vary between 0 and 88% among studies. However, the vast majority of the investigations revealed that in NEN SST4 is expressed at a low level and to a significantly lesser degree than the other SSTs^10,11,13–16,18,19,21–24,28–34^. In all cases, only cytoplasmic staining of cells was observed.

We recently generated and thoroughly characterised a novel monoclonal anti-SST4 antibody, 7H49L61. We demonstrated that this antibody is well suited both for immunocytochemistry and immunoblot analysis in basic research and for immunohistochemical staining of formalin-fixed, paraffin-embedded routine pathology samples^35^.

Given the limited and contradictory data on SST4 expression in NEN, we aimed to re-evaluate SST4 expression in a large panel of BP-NEN and GEP-NEN samples using the novel antibody 7H49L61 and to correlate the expression data with clinicopathological parameters.

## Materials and methods

### Antibody

A rabbit monoclonal antibody (7H49L61) that recognises the carboxyl-terminal tail of human SST4 was generated in collaboration with and obtained from Thermo Fisher Scientific (Waltham, MA, USA) and has been extensively characterised recently^35^. The peptide used to immunise rabbits was CQQEALQPEPGRKRIPLTRTTTF, corresponding to residues 366–388 of human SST4.

### Tumour specimens

We evaluated 722 tumour samples from 274 patients (in detail, 117 patients with only 1 sample, 49 patients with 2 samples, 45 patients with 3 samples, 28 patients with 4 samples, 13 patients with 5 samples, 7 patients with 6 samples, 4 patients with 7 samples, 2 patients with 8 samples, 3 patients with 9 samples, 1 patient with 10 samples, 2 patients with 12 samples, 1 patient with 14 samples, 1 patient with 16 samples, and 1 patient with 18 samples; 396 were primary tumour samples, 292 were metastatic samples, and for 34 samples, this information was not included in the patient records). From some patients, both primary and metastatic samples were available. Of the 274 tumours, 93 (34.0%) originated from the lungs (22 TCs, 27 ACs, 36 SCLCs, and 8 LCNECs), 18 (6.6%) from the stomach, 16 (5.8%) from the duodenum/jejunum, 58 (21.2%) from the ileum, 5 (1.8%) from the appendix, 11 (4.0%) from the colon, 15 (5.5%) from the rectum, and 48 (17.5%) from the pancreas. The localisation of 10 (3.6%) primary tumours was unknown. The samples were provided by the Institute of Pathology and Cytology Bad Berka (Bad Berka, Germany) and were surgically removed between 1998 and 2016 at the Department of General and Visceral Surgery, Zentralklinik Bad Berka (Bad Berka, Germany). Clinical data were gathered from patient records.

All procedures performed in this study involving human participants were in accordance with the 1964 Helsinki declaration and its later amendments. All experimental protocols, the patient information and the declaration of informed consent were approved by and permission was gained from the local ethics committee (Ethikkommission der Landesärztekammer Thüringen) for this retrospective analysis. Informed consent for the use of tissue samples for scientific purposes was obtained from all individual participants included in the study when entering the Theranostic Research Center, Zentralklinik Bad Berka, Bad Berka, Germany, and the Department of General and Visceral Surgery, Zentralklinik Bad Berka, Bad Berka, Germany. All data were analysed anonymously.

### Patient characteristics

The neuroendocrine tumours evaluated in the present investigation were obtained from 141 men (51.5% of cases) and 123 women (44.9%). For 10 patients (3.6%), the sex was unknown. The overall mean age of the patients at diagnosis was 58.5 years (median: 59.7 years, range: 12.1–83.9 years). Forty-seven of the (corresponding) primary tumours (17.1%) were classified as T1; 46 (16.8%) as T2; 58 (21.2%) as T3; and 24 (8.8%) as T4. For 99 patients (36.1%), the extent of the primary tumour was not reported. At diagnosis, lymph node metastases were present in 123 cases (44.9%), whereas 84 patients (30.6%) had none. For 67 patients (24.5%), the lymph node status was not known. Distant metastases were disclosed for 114 patients (41.6%), whereas 89 patients (32.5%) had no distant metastases at diagnosis. For 71 patients (25.9%), the presence of distant metastases was not reported. At diagnosis, 19 of 171 patients (11.1%) with GEP-NEN had Union for International Cancer Control stage I disease, 12 patients (7.0%) had stage II disease, 23 patients (13.5%) had stage III disease, and 102 patients (59.6%) had stage IV disease. For 15 GEP-NEN patients (8.8%), the disease stage was unknown. The disease stage was not determined for the 93 BP-NEN patients and the 10 patients with unknown tumour origin. Of the tumours, 108 (39.4%) had grade 1, 86 (31.4%) had grade 2, and 75 (27.4%) displayed grade 3 histology. Tumour grading was not reported for five (1.8%) patients. Of the tumours, 59 were functional tumours, i.e. tumours for which hormone-related symptoms such as gastrointestinal ulcers, diarrhoea, flushes or hypoglycaemia were reported in the patient files, and 87 were non-functional tumours, i.e. tumours for which the relevant symptoms were denied in the patient anamnesis. For 128 tumours this information was not included in the patient files. The median overall follow-up time was 53.3 months. At the end of the follow-up period, 155 patients were still living, and 85 patients had died. For 34 patients, no survival data were available. Among the patients who died, the median survival time was 27.9 months.

### Immunohistochemistry

From the paraffin blocks, 4-µm sections were prepared and floated onto positively charged slides. Immunostaining was performed by an indirect peroxidase labelling method as described previously^36^. Briefly, sections were deparaffinated, rehydrated by means of a descending ethanol series during which endogenous peroxidases were blocked by incubation of the samples for 45 min in 0.3% H_2_O_2_ in methanol, microwaved in 10 mM citric acid (pH 6.0) for 16 min at 600 W and incubated with antibody 7H49L61 (1:500) overnight at 4 °C. The primary antibody was detected using a biotinylated anti-rabbit IgG followed by incubation with peroxidase-conjugated avidin (Vector ABC “Elite” kit; Vector Laboratories, Burlingame, CA, USA). The binding of the primary antibody was visualised using 3-amino-9-ethyl carbazole in acetate buffer (BioGenex, San Ramon, CA, USA). Sections were rinsed, counterstained with Mayer’s haematoxylin, and mounted in Vectamount™ mounting medium (Vector Laboratories, Burlingame, CA, USA). Sections obtained from normal human cortex, pancreas (exocrine pancreas), and placenta were used as positive controls (Supplemental Fig. 1). For negative controls, 7H49L61 was either omitted, replaced by a rabbit IgG isotype control (ab125938; dilution: 1:100; Abcam, Cambridge, UK) or adsorbed for 2 h at room temperature with 10 µg/ml of the peptide used for immunisations (Supplemental Fig. 1).

**Figure 1:**
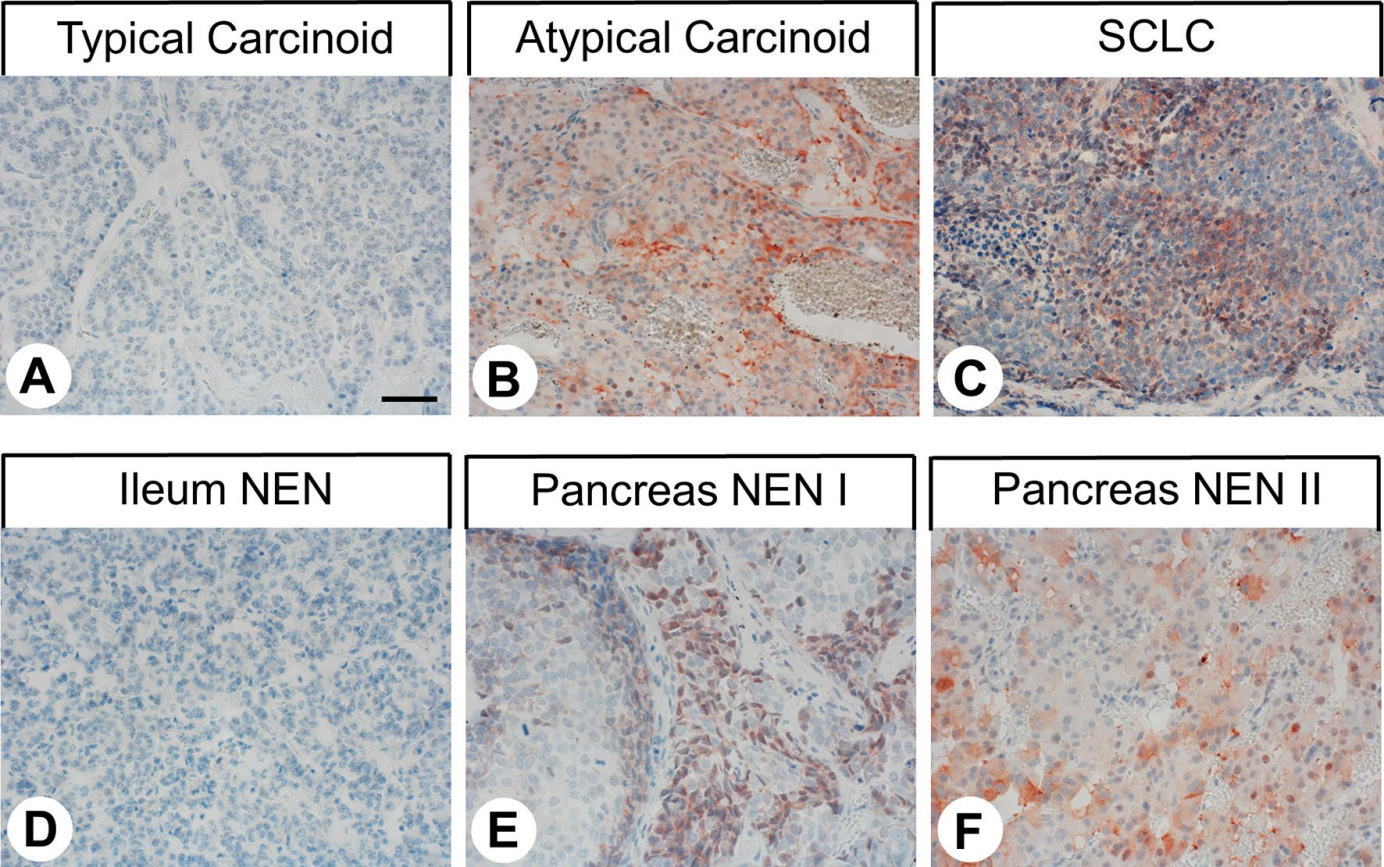
Representative staining patterns obtained in bronchopulmonary (**A**–**C**) and gastroenteropancreatic neuroendocrine neoplasms (**D**–**F**) using the antibody against somatostatin receptor 4 (SST4) 7H49L61, showing no (**A**, **D**) or moderate (**B**, **C**, **E**, **F**) SST4 expression intensity. Immunohistochemistry (red-brown colour), counterstaining with haematoxylin. Scale bar: 50 µm. NEN I, neuroendocrine neoplasm 1; NEN II, neuroendocrine neoplasm 2; SCLC, small-cell lung cancer.

The staining of SST4 in the tumours was assessed using the semiquantitative immunoreactivity score (IRS) according to Remmele and Stegner (1987)^37^. The percentage of positive tumour cells classified in five categories (no positive cells [0], < 10% positive cells [1], 10–50% positive cells [2], 51–80% positive cells [3], and > 80% positive cells [4]) was multiplied by the staining intensity classified in four categories (no staining [0], mild staining [1], moderate staining [2], and strong staining [3]). Thus, IRS values ranging from 0 to 12 were obtained. Only tumour samples with an IRS ≥ 3 were considered SST4-positive.

### Statistical analysis

For statistical analysis, SPSS 27.0.0.0 (IBM, Armonk, NY, USA) was used. Because the data were not normally distributed (according to a Kolmogorov–Smirnov test), Kruskal–Wallis test, Mann–Whitney U test, χ^2^ test, Kendall’s τ-b test, and Spearman’s rank correlation were performed. For survival analysis, the Kaplan–Meier method, with a log-rank test and a Breslow test, was used. For all analyses, *p* values ≤ 0.05 were considered significant.

## Results

### SST4 expression patterns

**Figure 2:**
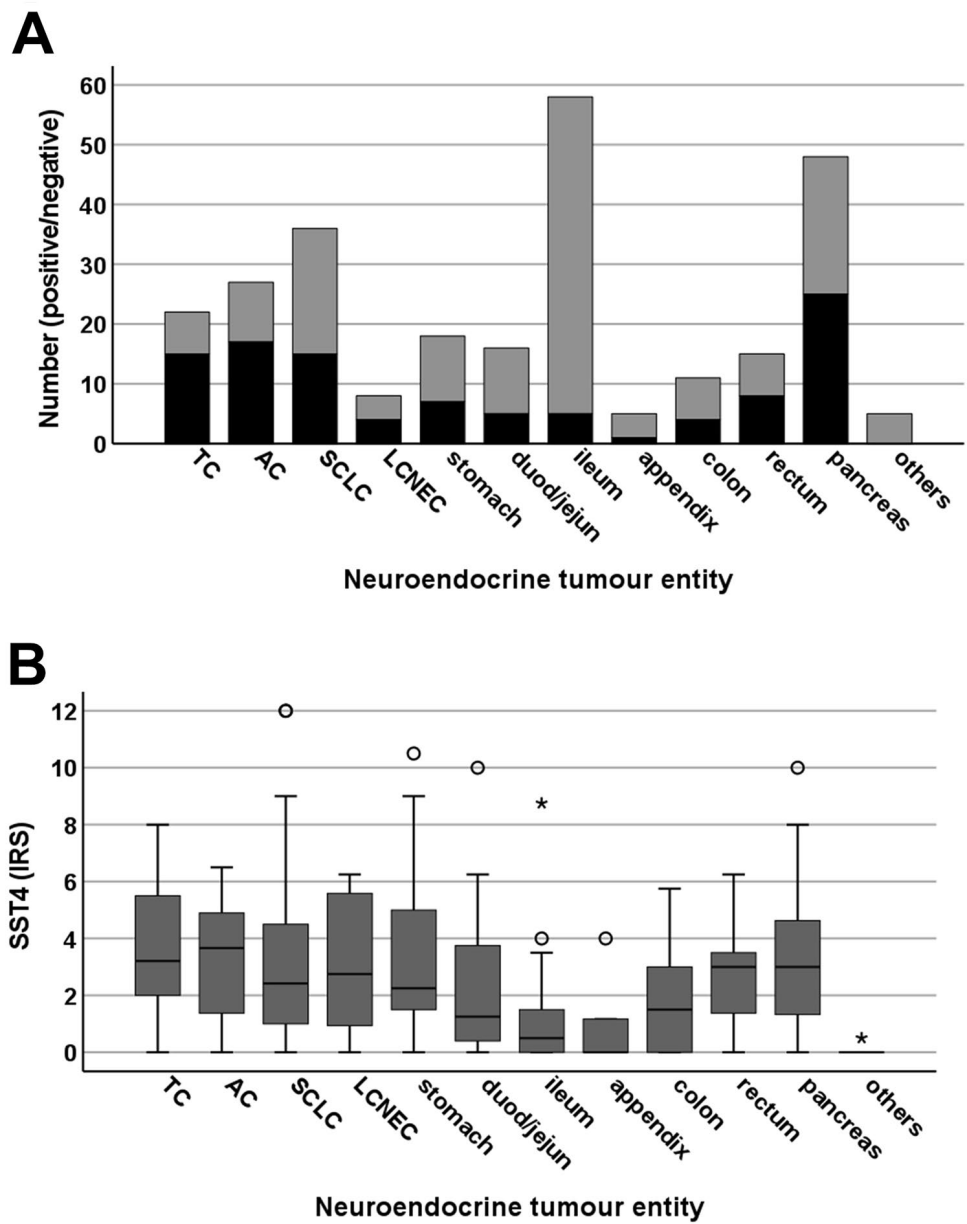
Somatostatin receptor 4 (SST4) expression in bronchopulmonary and gastroenteropancreatic neuroendocrine neoplasms by tumour entity and site of origin of the primary tumour. (**A**) Numbers of tumours positive (immunoreactivity score [IRS] ≥ 3) or negative (IRS < 3) for SST4. (**B**) Box plots of SST4 expression levels of the tumours, including those that were SST4-negative. Plots depict median values, upper and lower quartiles, minimum and maximum values, and outliers. For outliers, circles indicate mild outliers (1.5–3 interquartile range [IQR] from the nearest quartile), and asterisks indicate extreme outliers (> 3 IQR from the nearest quartile). AC, atypical carcinoid; duod, duodenum; jej, jejunum; LCNEC, large-cell neuroendocrine carcinoma of the lung; SCLC, small-cell lung cancer; TC, typical carcinoid.

Figure 1 shows representative images of BP-NEN and GEP-NEN samples stained with the anti-SST4 antibody 7H49L61. Figure 2A shows the number of SST4-positive (IRS ≥ 3) or SST4-negative (IRS < 3) BP-NEN and GEP-NEN tumours grouped by tumour entity and localisation of the primary tumour. In Fig. 2B, the respective expression levels are depicted, including those of SST4-negative tumours. In all cases, both cytoplasmic and membraneous staining of the cells was observed (Fig. 1). SST4 expression levels varied markedly between individual patients with the same tumour entity or primary tumour localisation, as indicated by the length of the respective boxes and whiskers in Fig. 2B; in some cases, even strong expression (IRS = 12) was observed. SST4 expression also varied distinctly between different samples from the same patient and within one tumour slide.

Across all BP-NEN and GEP-NEN tumours, 39.3% of the cases were SST4 positive (IRS ≥ 3). The median IRS, however, was only 2.0 (mean ± S.E.M.: 2.58 ± 0.15), corresponding to negative expression overall. There were, however, some differences in SST4 expression between BP-NEN and GEP-NEN tumours. Whereas 54.8% of BP-NEN tumours were SST4-positive and the overall median IRS was 3.0 (mean ± S.E.M.: 3.41 ± 0.27), corresponding to very low expression, only 32.2% of GEP-NEN cases were SST4-positive and the overall median IRS was 1.5 (mean ± S.E.M.: 2.22 ± 0.18), corresponding to no expression. The differences in SST4 positivity and SST4 expression levels between BP-NEN and GEP-NEN were significant (χ^2^ test: *p* < 0.001; Mann–Whitney test: *p* < 0.001). There were no differences in positivity rates and SST4 expression levels among the different BP-NEN entities, but SST4 expression varied significantly among GEP-NEN with different localisations of the primary tumour (χ^2^ test: *p* < 0.001; Kruskal–Wallis test: *p* < 0.001) (Fig. 2A and B). Tumours deriving from the ileum (median IRS: 0.50; mean IRS: 1.00) showed significantly lower SST4 expression than did those originating from the stomach (median IRS: 2.25; mean IRS: 3.55; Mann–Whitney test: *p* < 0.001), from the duodenum/jejunum (median IRS: 1.25; mean IRS: 2.42; Mann–Whitney test: *p* < 0.045), from the rectum (median IRS: 3.00; mean IRS: 2.69; Mann–Whitney test: *p* < 0.001), or from the pancreas (median IRS: 3.00; mean IRS: 3.17; Mann–Whitney test: *p* < 0.001). Similarly, tumours originating from the appendix (median IRS: 0.00; mean IRS: 1.03) displayed significantly lower IRS values than did those deriving from the stomach (Mann–Whitney test: *p* = 0.046) or from the pancreas (Mann–Whitney test: *p* = 0.029).

### Correlations with clinical data

Because expression and prognostic impact of the other four somatostatin receptors, especially SST1 and SST2, differ between BP-NEN and GEP-NEN^17,18,23^, we analysed correlations between SST4 expression and clinical data separately for the two tumour types.

**Figure 3:**
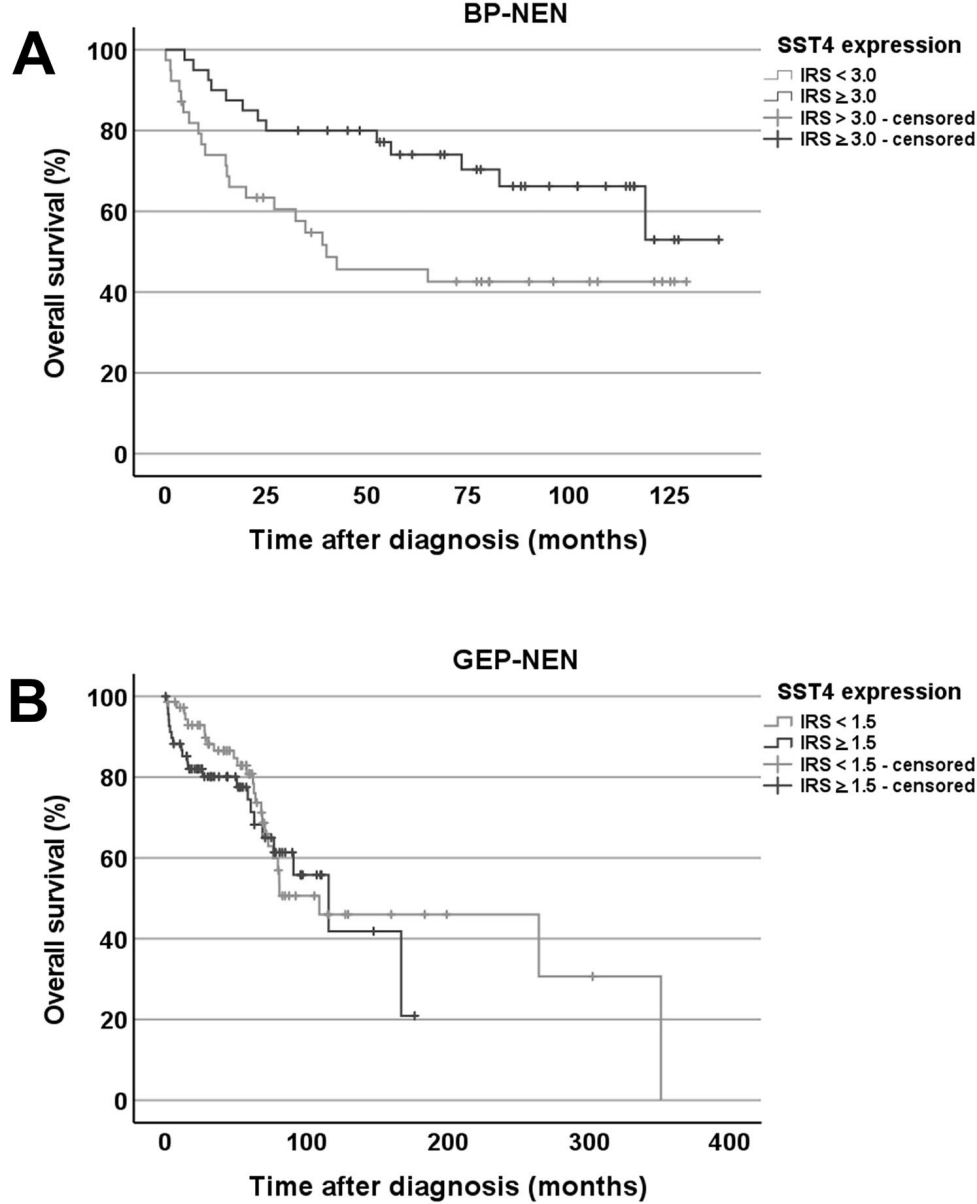
Overall survival of patients with (**A**) bronchopulmonary neuroendocrine neoplasm (BP-NEN) or (**B**) gastroenteropancreatic neoplasm (GEP-NEN) by somatostatin receptor 4 (SST4) expression of their tumours. The median immunoreactivity scores (IRSs) for all BP-NEN tumours of 3.0 and for all GEP-NEN tumours of 1.5 were set as the cut-off values for discrimination between high or low SST4 expression. (**A**) Log-rank test: *p* = 0.021; Breslow test: *p* = 0.007. (**B**) Log-rank test: *p* = 0.530; Breslow test: *p* = 0.245.

In BP-NEN, SST4 expression did not vary with sex, age, smoking status, or overall survival of the patients; tumour size; the presence of lymph node or distant metastases at diagnosis; or tumour stage or grade. No differences were observed in SST4 IRS between primary tumours and metastases or between tumours from patients who were still alive at the end of the observation period and those who died from tumour-related causes. There was, however, a significant negative correlation between SST4 expression and the expression of the proliferation marker Ki-67 (Table 1). This finding was corroborated by Kaplan–Meier analyses. When using IRS ≥ 3 (the overall median IRS of BP-NEN) as the cut-off value, patients with higher receptor expression had significantly better outcomes than those with lower or no SST4 expression (log-rank test: *p* = 0.021; Breslow test: 0.007; Fig. 3A).

**Table 1:** Correlations between the expression intensities of somatostatin receptor 4 (SST4), the four other SSTs, CXC motif chemokine receptor 4 (CXCR4), chromogranin A (CgA), Ki-67, and programmed cell death 1 ligand 1 (PD-L1) in bronchopulmonary neuroendocrine neoplasms (calculated using the mean receptor immunoreactivity scores for each patient; n = 93).

		SST1	SST2	SST3	SST5	CXCR4	CgA	Ki-67	PD-L1
SST4	r	0.076	− 0.173	− 0.035	0.004	− 0.015	0.155	**− 0.213**	0.129
	*p*	0.480	0.107	0.744	0.967	0.894	0.156	**0.044**	0.227
SST1	r		0.026	0.053	**0.525**	**− 0.285**	**0.303**	**− 0.540**	**− 0.240**
	*p*		0.809	0.616	**< 0.001**	**0.008**	**0.004**	**< 0.001**	**0.025**
SST2	r			− 0.015	− 0.028	− 0.069	0.043	0.021	− 0.068
	*p*			0.886	0.788	0.529	0.687	0.845	0.532
SST3	r				**0.270**	0.087	0.001	0.014	0.077
	*p*				**0.009**	0.426	0.992	0.892	0.478
SST5	r					− 0.107	0.154	**− 0.303**	− 0.196
	*p*					0.325	0.152	**0.003**	0.069
CXCR4	r						**− 0.537**	**0.628**	**0.319**
	*p*						**< 0.001**	**< 0.001**	**0.003**
CgA	r							**− 0.659**	**− 0.372**
	*p*							**< 0.001**	**< 0.001**
Ki-67	r								**0.347**
	*p*								**< 0.001**

Significant correlations (*p* < 0.05) are marked in bold; r: Spearman correlation coefficient.

Similarly, in GEP-NEN, no impact of sex, age, tumour size, lymph node status, presence of distant metastases, or tumour stage on SST4 expression was detected, and no difference in SST4 expression was observed between primary tumours and metastases or between tumours from patients who were still living at the end of the observation period and those who had died. However, functional tumours had significantly lower IRS values compared to non-functional tumours (mean ± S.E.M.: functional: 1.43 ± 0.25; non-functional: 2.21 ± 0.22; Mann–Whitney test: *p* = 0.007). Furthermore, SST4 expression in the tumours negatively correlated with serum serotonin values (rsp = − 0.322; *p* = 0.023), but not with serum chromogranin A (CgA) values (rsp = − 0.106; *p* = 0.380). Additionally, positive correlations between SST4 expression and tumour grade (Kendall’s τ-b test: *p* = 0.004) and Ki-67 index (Table 2) and a negative interrelationship with overall survival (rsp = − 0.376; *p* = 0.008) were observed. In the respective Kaplan–Meier analyses, when using the overall median IRS of GEP-NEN of 1.5 as the cut-off value between groups, however, no such interrelationship was detected (log-rank test: *p* = 0.530; Breslow test: *p* = 0.245; Fig. 3B).

**Table 2:** Correlations between the expression intensities of somatostatin receptor 4 (SST4), the four other SSTs, CXC motif chemokine receptor 4 (CXCR4), chromogranin A (CgA), Ki-67, and programmed cell death 1 ligand 1 (PD-L1) in gastroenteropancreatic neuroendocrine neoplasms (calculated using the mean receptor immunoreactivity scores for each patient; n = 171).

		SST1	SST2	SST3	SST5	CXCR4	CgA	Ki-67	PD-L1
SST4	r	**0.233**	0.150	**0.172**	**0.189**	0.125	**0.181**	**0.257**	**0.399**
	*p*	**0.004**	0.053	**0.026**	**0.015**	0.108	**0.019**	**< 0.001**	**< 0.001**
SST1	r		0.023	**0.317**	**0.397**	**0.259**	− 0.010	**0.192**	**0.261**
	*p*		0.763	**< 0.001**	**< 0.001**	**< 0.001**	0.893	**0.013**	**< 0.001**
SST2	r			**0.236**	0.141	0.013	**0.298**	− 0.052	**0.242**
	*p*			**0.002**	0.068	0.865	**< 0.001**	0.500	**0.002**
SST3	r				**0.294**	0.125	**0.248**	0.050	**0.255**
	*p*				**< 0.001**	0.108	**0.001**	0.520	**< 0.001**
SST5	r					**0.230**	− 0.099	**0.290**	**0.293**
	*p*					**0.003**	0.201	**< 0.001**	**< 0.001**
CXCR4	r						**− 0.167**	**0.260**	0.081
	*p*						**0.031**	**< 0.001**	0.303
CgA	r							− 0.119	**0.229**
	*p*							0.124	**0.003**
Ki-67	r								**0.313**
	*p*								**< 0.001**

Significant correlations (*p* < 0.05) are marked in bold; r: Spearman correlation coefficient.

### Correlations with other tumour markers

We also analysed interrelationships between SST4 expression and the expression of the other four SST subtypes as well as the expression of other receptors of or markers for neuroendocrine tumours, such as the chemokine receptor CXCR4, CgA, and programmed cell death 1 ligand 1 (PD-L1), in BP-NEN and GEP-NEN. These expression data were obtained for a subset of the present samples from earlier studies using exactly the same staining protocol and rating method^18,23,38^ (detailed information regarding the clones, epitopes, and dilutions of the antibodies is given in Supplemental Table 1). Whereas no correlations between SST4 and the other receptors or markers were noted in BP-NEN (Table 1), positive interrelationships between SST4 and SST1, SST3, SST5, CgA, and PD-L1 were observed in GEP-NEN (Table 2).

In addition, and similar to previous observations^23,38^, in BP-NEN significant positive correlations between the IRS of SST1 and that of SST5 or of CgA and negative associations between the expression of SST1 and that of CXCR4, Ki-67 or PD-L1 were observed. Additionally, a positive interrelationship between SST3 and SST5 was noted. Negative associations were observed between the expression levels of SST5 and Ki-67, between those of CXCR4 and CgA and between those of CgA and Ki-67 or PD-L1, but positive associations were noted between the expression levels of CXCR4 and Ki-67 or PD-L1 and between the presence of Ki-67 and that of PD-L1 (Table 1).

In GEP-NEN, as a supplementary finding and as partially reported before^18^, positive correlations were observed between SST1 and SST3, SST5, CXCR4, Ki-67 or PD-L1 expression; between SST2 and SST3, CgA or PD-L1 expression; between SST3 and SST5, CgA or PD-L1 expression; between SST5 and CXCR4, Ki-67 or PD-L1 expression; between CXCR4 and Ki-67 expression; between CgA and PD-L1 expression; and between Ki-67 and PD-L1 expression, whereas a negative association was noted between CXCR4 and CgA expression (Table 2).

## Discussion

In our study, 39.3% of tumours were SST4-positive, including 54.8% of BP-NEN and 32.2% of GEP-NEN. This corresponds well with the findings of most previous reports^10,11,13–16,18,19,21–24,28–34^, but unlike the earlier immunohistochemical studies, our investigation demonstrated for the first time cytoplasmic as well as membraneous staining of tumour cells. In spite of this, the overall median IRS across tumours was only 2.0, and, thus, below the threshold set for receptor positivity. Therefore, SST4 expression seems to be negligible in NEN overall. There were some differences in SST4 positivity rates and SST4 expression levels between BP-NEN and GEP-NEN and also between GEP-NEN tumours with different sites of origin. Nevertheless, the highest median IRS observed across all NEN entities and sites of origin was noted in AC and amounted to only 3.7, corresponding to low expression.

In the present study, we observed a significant negative correlation between SST4 expression and Ki-67 index and significantly better outcomes in patients with higher receptor expression than in those with lower or no SST4 expression for BP-NEN. This corresponds well to literature data showing that SST4 expression decreases with increasing malignancy from AC to SCLC in BP-NEN^22^. Conversely, in GEP-NEN, we noted positive correlations between SST4 expression and tumour grade as well as Ki-67 index, which also fit well with previous reports that SST4 mRNA expression increases with tumour grade in GEP-NEN^33^. Functional GEP-NEN have been reported to be associated with better patient outcomes^39–42^, which matches well with our result that functional GEP-NENs exhibited significantly lower SST4 expression levels than non-functional tumours. Overall, there seems to be an opposite relationship between SST4 expression and patient outcomes in BP-NEN versus GEP-NEN tumours. Very recently, similar divergent observations have been made for G protein–coupled oestrogen receptor expression in these tumours^43^.

## Conclusion

In contrast to other SSTs, SST4 expression in both BP-NEN and GEP-NEN is negligible and of no diagnostic or therapeutic relevance.

## Supplementary Information

Supplementary Information 1.

Supplementary Information 2.

## Data Availability

All data generated during this study are included in this published article. The amino acid sequence of the receptor against which the antibody was raised is publicly available through the Uniprot database (https://www.uniprot.org/uniprotkb/P31391/entry).

## Abbreviations

AC: Atypical carcinoid
BP-NEN: Bronchopulmonary neuroendocrine neoplasm
CgA: Chromogranin A
CXCR4: CXC motif chemokine receptor 4
GEP-NEN: Gastroenteropancreatic neuroendocrine neoplasm
IRS: Immunoreactivity score
Ki-67: Proliferation marker
LCNEC: Large-cell neuroendocrine carcinoma of the lung
NEN: Neuroendocrine neoplasm
PD-L1: Programmed cell death 1 ligand 1
rsp: Spearman correlation coefficient
SCLC: Small-cell lung cancer
SST: Somatostatin receptor
TC: Typical carcinoid
