# Supplementary figures and images for: Reassessment of somatostatin receptor SST4 expression in bronchopulmonary and gastroenteropancreatic neuroendocrine neoplasms using the novel rabbit monoclonal anti-human SST4 antibody 7H49L61
Source: Sci Rep. 2022 Aug 30;12:14722. doi: 10.1038/s41598-022-19014-w (PMC9428033; doi:10.1038/s41598-022-19014-w)

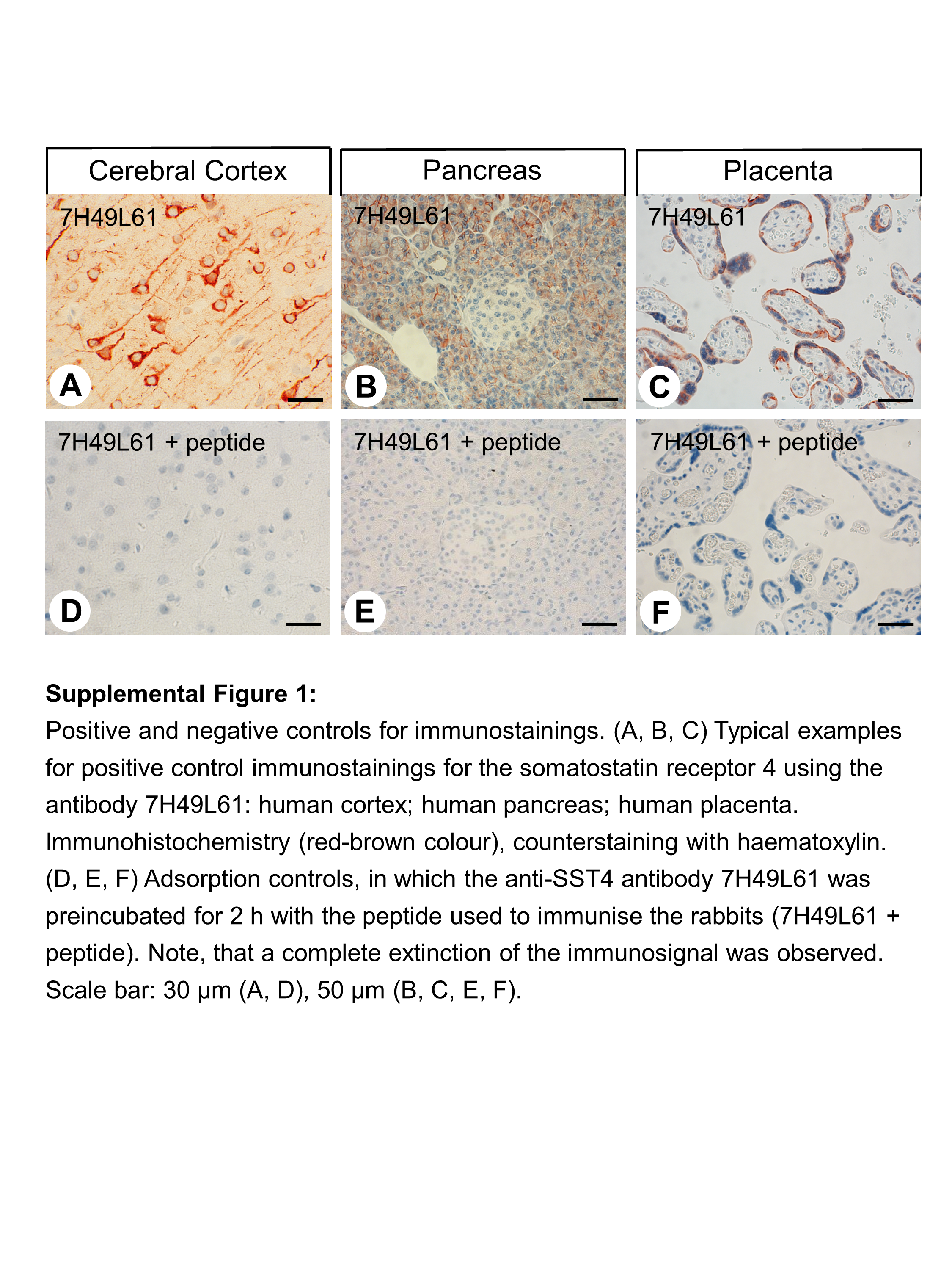

Supplement: Supplementary file 1 — Supplementary Information 1. [file 41598_2022_19014_MOESM1_ESM.tif]
