# Supplementary material for: Reassessment of somatostatin receptor SST4 expression in bronchopulmonary and gastroenteropancreatic neuroendocrine neoplasms using the novel rabbit monoclonal anti-human SST4 antibody 7H49L61
Source: Sci Rep. 2022 Aug 30;12:14722. doi: 10.1038/s41598-022-19014-w (PMC9428033; doi:10.1038/s41598-022-19014-w)
Supplement: Supplementary file 2 — Supplementary Information 2. [file 41598_2022_19014_MOESM2_ESM.docx]

**Supplemental Table 1:** Antibodies used for immunohistochemical stainings in the present investigation and in previous studies [18, 23, 38].

| **Antibody** | **Clone** | **Type** | **Epitope** | **Supplier** | **Dilution** |
| --- | --- | --- | --- | --- | --- |
| **SST1** | UMB-7 | rabbit monoclonal | ENLESGGVFRNGTCTSRITTL  (residues 377-391) | Abcam Cambridge, UK | 1:25 |
| **SST2** | UMB-1 | rabbit monoclonal | ETQRTLLNGDLQTSI  (residues 335-369) | Abcam Cambridge, UK | 1:10 |
| **SST3** | UMB-5 | rabbit monoclonal | QLLPQEASTGEKSSTMRISYL  (residues 398-418) | Abcam Cambridge, UK | 1:20 |
| **SST4** | 7H49L61 | rabbit monoclonal | CQQEALQPEPGRKRIPLTRTTTF  (residues 366-388) | Thermo Fisher Scientific, Waltham, MA, USA | 1:500 |
| **SST5** | UMB-4 | rabbit monoclonal | QEATPPAHRAAANGLMQTSKL  (residues 344-364) | Abcam Cambridge, UK | 1:10 |
| **CXCR4** | UMB-2 | rabbit monoclonal | KGKRGGHSSVSTESESSSFHSS  (residues 338-359) | Abcam Cambridge, UK | 1:2 |
| **PD-L1** | 73-10 | rabbit monoclonal | CLGVALTFIFRLRKGRMMDVKKCGIQDTNSKKQSDTHLEET  (residues 250-290) | Abcam Cambridge, UK | 1:1000 |
| **CgA** | LK2H10 | mouse monoclonal |  | BioLogo, Kronshagen, Germany | 1:50 |
| **Ki-67** | MIB-1 | mouse monoclonal |  | DAKO, Carpintera, CA, USA | 1:50 |
